# Unveiling the Solvent Effect: DMSO Interaction with Human Nerve Growth Factor and Its Implications for Drug Discovery

**DOI:** 10.3390/molecules30143030

**Published:** 2025-07-19

**Authors:** Francesca Paoletti, Tjaša Goričan, Alberto Cassetta, Jože Grdadolnik, Mykola Toporash, Doriano Lamba, Simona Golič Grdadolnik, Sonia Covaceuszach

**Affiliations:** 1 Institute of Crystallography-C.N.R.-Trieste Outstation, 34149 Trieste, Italy; francesca.paoletti@cnr.it (F.P.); alberto.cassetta@cnr.it (A.C.); mykolatoporash@cnr.it (M.T.); doriano.lamba@cnr.it (D.L.); 2 Laboratory for Molecular Structural Dynamics, Theory Department, National Institute of Chemistry, 1001 Ljubljana, Slovenia; tjasa.gorican@ki.si (T.G.); joze.grdadolnik@ki.si (J.G.); simona.grdadolnik@ki.si (S.G.G.); 3 Interuniversity Consortium “Biostructures and Biosystems National Institute”, 00136 Rome, Italy

**Keywords:** Nerve Growth Factor (NGF), dimethyl sulfoxide (DMSO), drug discovery, Nuclear Magnetic Resonance (NMR), Fourier Transform Infrared (FT-IR) spectroscopy, Differential Scanning Fluorimetry (DSF), Grating-Coupled Interferometry (GCI), solvent effect, Protenix, HADDOCK

## Abstract

**Background:** The Nerve Growth Factor (NGF) is essential for neuronal survival and function and represents a key therapeutic target for pain and inflammation-related disorders, as well as for neurodegenerative diseases. Small-molecule antagonists of human NGF (hNGF) offer advantages over monoclonal antibodies, including oral availability and reduced immunogenicity. However, their development is often hindered by solubility challenges, necessitating the use of solvents like dimethyl sulfoxide (DMSO). This study investigates whether DMSO directly interacts with hNGF and affects its receptor-binding properties. **Methods:** Integrative/hybrid computational and experimental biophysical approaches were used to assess DMSO-NGF interaction by combining machine-learning tools and Nuclear Magnetic Resonance (NMR), Fourier Transform Infrared (FT-IR) spectroscopy, Differential Scanning Fluorimetry (DSF) and Grating-Coupled Interferometry (GCI). These techniques evaluated binding affinity, conformational stability, and receptor-binding dynamics. **Results:** Our findings demonstrate that DMSO binds hNGF with low affinity in a specific yet non-disruptive manner. Importantly, DMSO does not induce significant conformational changes in hNGF nor affect its interactions with its receptors. **Conclusions:** These results highlight the importance of considering solvent–protein interactions in drug discovery, as these low-affinity yet specific interactions can affect experimental outcomes and potentially alter the small molecules binding to the target proteins. By characterizing DMSO-NGF interactions, this study provides valuable insights for the development of NGF-targeting small molecules, supporting their potential as effective alternatives to monoclonal antibodies for treating pain, inflammation, and neurodegenerative diseases.

## 1. Introduction

The Nerve Growth Factor (NGF), the first identified member of the neurotrophin (NT) family, was initially discovered as a vital survival factor for the development and differentiation of sympathetic and sensory neurons during embryogenesis [1,2]. Subsequent research revealed that NGF exerts a wide range of functions in both neural and non-neural systems in adulthood via interactions with two types of receptors: the Trk tyrosine kinase (TrkA), which is specific for NGF, and the low-affinity pan-neurotrophin receptor p75^NTR^, which binds all NTs with comparable affinity [3,4]. These include promoting differentiation, supporting neuronal survival, facilitating synaptogenesis, and regulating synaptic plasticity, highlighting its multifaceted role across various biological processes [5].

NGF is essential for nociceptive sensory neurons, and its dysregulation has been linked to chronic pain, inflammation, certain neurodegenerative diseases, tumorigenesis, and cancer pain [6,7,8], making it a target for novel analgesics aimed at disrupting human NGF (hNGF) signalling pathways [9]. Targeting hNGF holds substantial therapeutic potential for managing conditions such as chronic nociceptive and neuropathic pain states, osteoarthritis, chronic low back pain, interstitial cystitis, and cancer pain [10,11].

Additionally, hNGF is involved in the pathogenesis of various immune diseases, including chronic arthritis [12].

hNGF has thus emerged as a significant target for drug development, particularly for neurological and inflammatory conditions. However, due to the many diverse physiological roles of hNGF, including in immune function and neuroprotection, care should be taken to avoid the long-term adverse effects of its pharmacological applications [13].

Therapeutic strategies targeting hNGF include both small molecules and monoclonal antibodies like tanezumab, which block hNGF interaction with TrkA and p75^NTR^ receptors to treat chronic pain conditions [14]. Despite showing promise, these therapies—tanezumab, fulranumab, and fasinumab—have faced setbacks due to safety concerns, particularly joint-related adverse events, which have limited their clinical progress [15,16].

For these reasons, small-molecule antagonists of hNGF are emerging as a promising area of research, primarily aimed at modulating pain pathways and addressing inflammation-associated conditions. These antagonists prevent hNGF from binding to its receptors, TrkA and p75^NTR^, disrupting their downstream signalling pathways involved in pain and inflammation [17]. Certain small molecules can inhibit hNGF synthesis or induce conformational changes that render hNGF incapable of receptor binding [18].

One promising avenue involves small molecules derived from neurotrophic proteins, which can mimic or antagonize hNGF actions selectively [19]. Another approach involves the development of peptidomimetics to competitively inhibit receptor binding [20].

Small molecules offer distinct benefits over monoclonal antibodies: (i) an advantage in the delivery because they can potentially be administered orally, unlike monoclonal antibodies that require epidural administration; (ii) they are generally more affordable to be synthesized; (iii) they are less likely to trigger immune responses.

The screening to identify new small molecules or chemical fragments to develop hNGF antagonists with optimized binding and selectivity has the great advantage of leveraging integrative advanced computational methods and structural biology tools. These molecules could also be employed as complementary therapies when combined with anti-inflammatory drugs or physical therapy. Thus, small-molecule antagonists of hNGF represent a promising avenue to target hNGF-related pathologies, including pain management. Despite these benefits, challenges are present, including achieving specificity without off-target effects by designing molecules that selectively target hNGF without affecting other neurotrophins or signalling pathways.

Many small molecules exhibit poor water solubility, particularly in the early stages of development when the optimization of their physicochemical properties may not yet be complete. To address this issue, these molecules are often dissolved in organic solvents like dimethyl sulfoxide (DMSO) for in vitro and in vivo studies. While DMSO is a widely used solvent due to its broad solubility profile, its low chemical reactivity, and its relatively low toxicity; it poses specific challenges when studying interactions between small molecules and targets like hNGF.

DMSO is an amphipathic molecule with polar and nonpolar regions, making it a versatile solvent. Although the percentage of DMSO typically used in experimental procedures for ligand–protein binding studies is not high enough to disrupt the three-dimensional structure in proteins, DMSO’s versatility may allow it to interact with proteins. For example, DMSO can disrupt hydrophobic interactions and hydrogen bonding in proteins, potentially leading to conformational changes. Different effects of DMSO have been reported for various proteins. For example, DMSO has been shown to act either as a stabilizer or a denaturant of proteins, depending on the protein and experimental conditions [21], or to influence the relative binding affinities of protein–ligand complexes [22]. Studies have shown that DMSO can induce subtle changes in the viscosity of DMSO–water mixtures, which could affect protein dynamics measurements [23], as well as alter binding affinities in ligand–protein assays [24].

It cannot be excluded a priori that similar alterations might happen in hNGF antagonist binding studies, affecting hNGF’s ability to bind to its receptors and thereby influencing experimental outcomes.

Furthermore, DMSO may directly bind to hNGF’s ligand-binding sites or other hydrophobic pockets, mimicking or blocking the effects of the small molecule being studied. This is particularly relevant in studies involving structure–activity relationships (SARs), as DMSO interference may obscure the true nature of ligand binding.

While DMSO is an indispensable solvent in early-stage drug discovery, its potential to interact with hNGF and influence the binding of small molecules cannot be ignored. Understanding these limitations is crucial for the accurate characterization of hNGF antagonists and their translation into therapeutic candidates.

Nevertheless, it is important to underline that DMSO does not invariably interfere with small-molecule behaviour. Several studies, including the recent literature [25], demonstrate that many small-molecule–target complexes retain their overall structural integrity, and the drug interaction profiles are unchanged even at relatively high DMSO concentrations. Thus, while caution is warranted, DMSO’s effects should be evaluated on a case-by-case basis rather than assumed.

In order to address these important issues, we combined experimental biophysical approaches such as FT-IR, DSF, GCI, and NMR together with computational studies in order to investigate the specific effects of DMSO on hNGF. Results demonstrate that DMSO does not induce secondary structure changes but can bind hNGF with low affinity in a specific manner, highlighting a critical consideration for the development of new hNGF-targeting fragments or molecules. As research progresses, small-molecule antagonists of hNGF hold great promise for treating pain and other hNGF-related conditions, offering a cost-effective and patient-friendly alternative to monoclonal antibodies. Thus, it should be taken into consideration the interfering/competing effect that DMSO might have in the interpretation of hNGF–ligand experiments.

## 2. Results

### 2.1. DMSO Does Not Induce Significant Conformational Changes in hNGF

**Figure 1:**
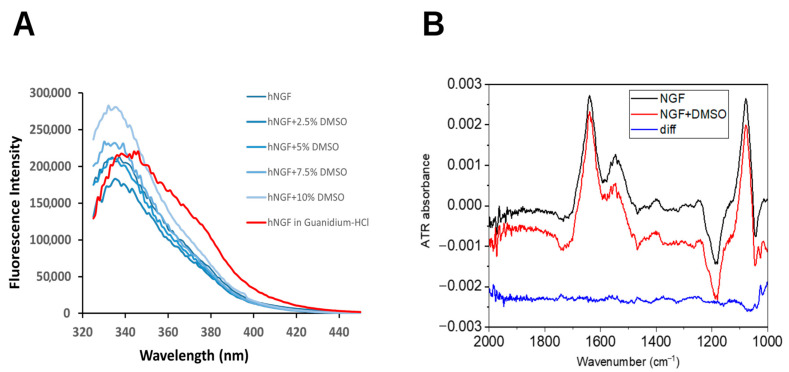
Impact of DMSO on hNGF structure. (**A**) Intrinsic fluorescence emission spectra of hNGF in the presence of increasing DMSO concentrations (from 2.5% to 10%, from dark to light blue). hNGF without DMSO: dark blue line. hNGF in 6 M Guanidine hydrochloride: red curve. (**B**) FT-IR spectra of hNGF in the presence and absence of DMSO. Blue spectrum: the difference between the red and black spectrum (downshifted for clarity). DMSO spectrum in Supplementary Figure S1.

Intrinsic fluorescence measurements of hNGF in the presence of DMSO concentrations up to 10% did not show significant shifts in the emission maximum, thus suggesting an absence of the denaturation process of the protein. However, a change in emission maximum intensity was detected, which is suggestive of a binding effect on hNGF (Figure 1A).

Moreover, the FT-IR spectra of hNGF were collected in the presence of an increasing DMSO concentration, ranging from 0% to 8%, which reflects the typical conditions used for ligand-based studies). No detectable changes in the amide I band (a sensitive indicator of protein secondary structure) were observed up to 0.8% DMSO (Figure 1B). Beyond this concentration, an apparent distortion of the amide I band was detected. However, a careful investigation of the FT-IR spectra of DMSO alone ruled out any specific effect of DMSO on the hNGF secondary structure. Indeed, a similar distortion was also observed at the same concentration, in the absence of protein. This distortion is most likely due to a change in the solvation properties at higher DMSO concentrations.

These findings support the conclusion that DMSO does not alter hNGF’s secondary structure within the tested concentration range.

### 2.2. DMSO Specifically Interacts with hNGF at Low Affinity Without Affecting Its Receptor Binding

Building on the FT-IR results, highlighting that under the tested experimental conditions, DMSO does not perturb the native secondary structure of hNGF, DSF measurements were carried out, aiming to qualitatively monitor the thermal stability.

**Figure 2:**
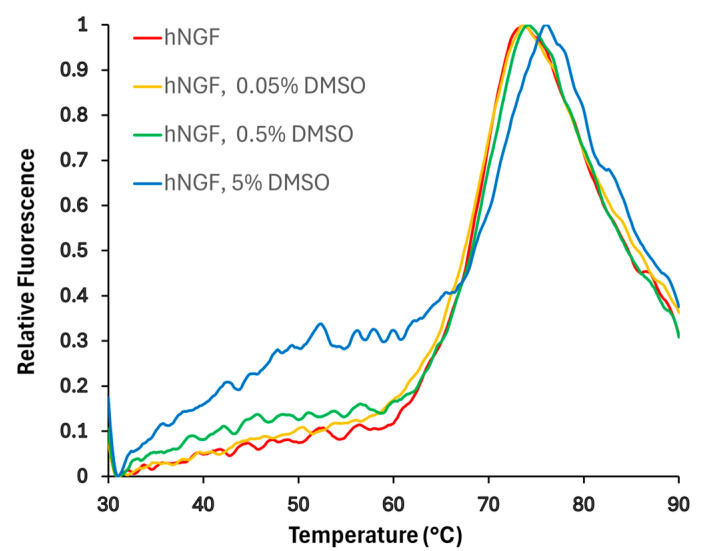
Impact of DMSO concentration on hNGF melting curves obtained by DSF. Normalized curves are shown. The buffer conditions were 50 mM sodium phosphate, pH 6.6, and 50 mM NaCl. The effect of three different DMSO concentrations was tested, obtaining: Tm: 67.9 ± 0.1 °C (0% DMSO); 67.9 ± 0.5 °C (0.05% DMSO); 67.9 ± 0.4 °C (0.5% DMSO); 69.1 ± 0.9 °C (0.5% DMSO).

A detectable change in hNGF’s melting temperature (Tm), when dissolved in buffer containing up to 5% DMSO (Figure 2), has been observed with a Tm shift from 67.9 ± 0.1 °C to 69.1 ± 0.9 °C. Notably, the impact of DMSO on hNGF melting temperature is both pH and salt concentration-dependent (Supporting Figure S2), with the larger DMSO effect on Tm reported for the combination of pH 7.2 and 50 mM NaCl. This observation suggests that DMSO interacts with hNGF, potentially influencing its biophysical properties.

To quantitatively characterize this interaction, we conducted detailed Grating-Coupled Interferometry (GCI) experiments. These studies aimed at investigating the binding kinetics and affinity of the DMSO-hNGF interaction while also evaluating its potential impact on hNGF’s receptor-binding dynamics and broader experimental implications.

**Figure 3:**
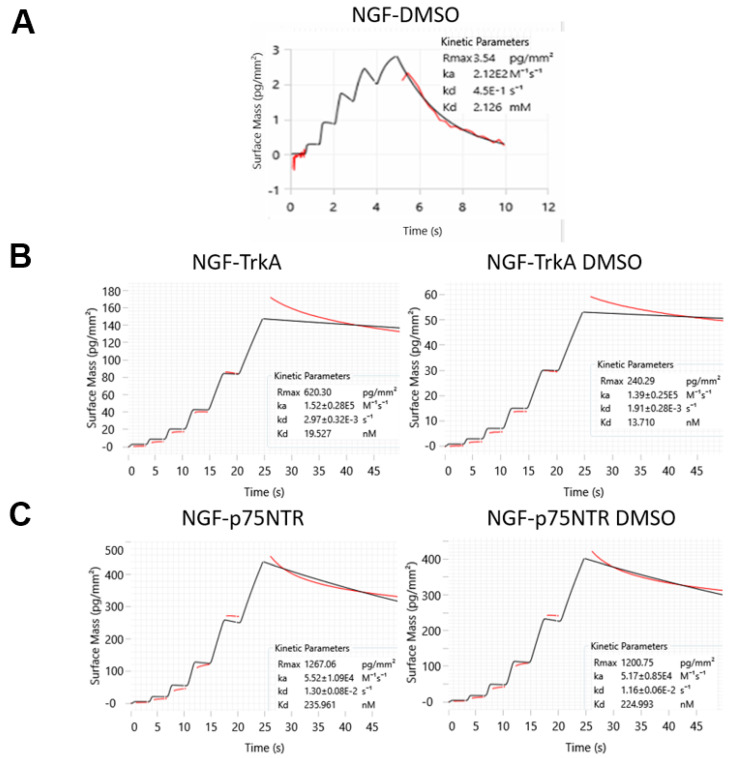
WaveRAPID kinetic and affinity data obtained using the Creoptix WAVE-delta system: (**A**) for hNGF-DMSO interaction and to compare hNGF binding to its receptors; (**B**) TrkA and (**C**) p75^NTR^, in the absence and presence of DMSO. All the quality assessments were fulfilled: χ^2^ (1.29 ± 0.15; 1.57 ± 0.34, 1.08 ± 0.22. 1.66 ± 0.28, and 1.13 ± 0.19 for hNGF-DMSO, hNGF-TrkA, hNGF-TrkA DMSO, hNGF- p75^NTR^, and hNGF-p75^NTR^ DMSO, respectively); parameter errors and residual plots were acceptable, the sensorgrams had sufficient curvatures, and the kinetic constant k_d_ was within a measurable range.

Representative sensorgrams from GCI analyses (Figure 3A) revealed that DMSO binds specifically to hNGF, with a k_a_ of 2.12 ± 0.25 10^2^ M^−1^ s^−1^ and a k_d_ of 4.50 ± 0.44 10^−1^ s^−1^, resulting in a calculated dissociation constant (K_D_) of approximately 2.1 ± 0.2 mM. In spite of a low-affinity binding constant, this interaction is significant because it highlights that DMSO, even at relatively low concentrations (e.g., 0.05%, which corresponds to approximately 7 mM DMSO), can saturate hNGF binding sites to ca. 80%, potentially leading to inaccurate protein–ligand experimental binding results. It is worth noting that the DMSO molar concentration is substantially higher than that of typical low-affinity small-molecule ligands. Hence, this range of DMSO concentration raises important concerns about the potential for false positives in experiments, especially when assessing weak binders or low-affinity interactions, as commonly encountered in Fragment-Based Drug Discovery (FBDD) studies.

Furthermore, to assess the potential impact of DMSO on hNGF’s receptor-binding dynamics, we conducted a series of GCI experiments. As shown in Figure 3B, the presence of DMSO does not significantly alter hNGF’s interaction with its receptors, TrkA or p75^NTR^, either in terms of thermodynamic (binding constant) or kinetic fingerprints. Namely, the determined association and dissociation rates remained within the same range, indicating that DMSO does not interfere with hNGF’s ability to engage its physiological partners. These findings suggest that hNGF retains its native binding properties even in the presence of DMSO, reinforcing the idea that DMSO interaction is weak and does not induce conformational changes that could affect the receptor’s recognition.

### 2.3. Structural Determinants of hNGF-DMSO Interaction

**Figure 4:**
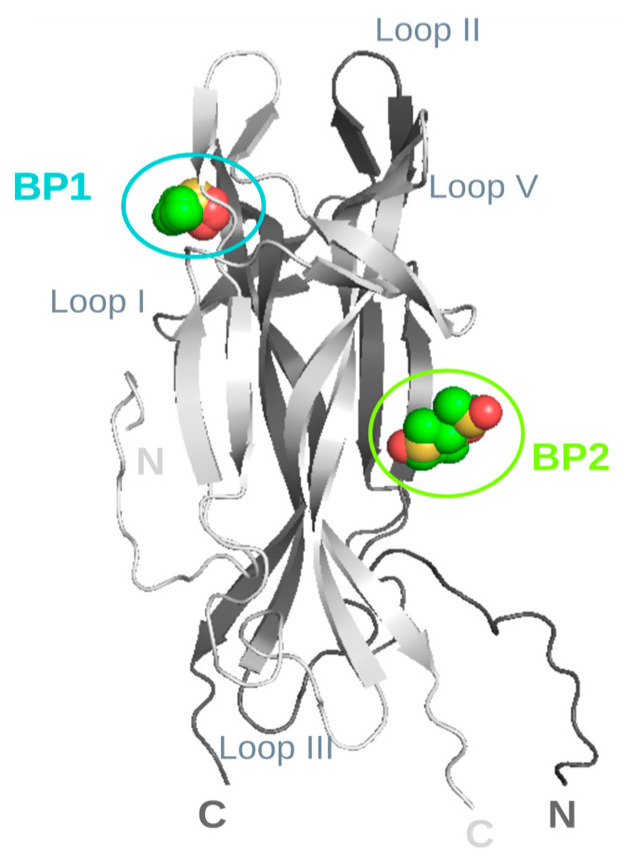
Ab initio models of hNGF-DMSO interaction. The identified binding pockets are labelled as BP1 (in cyan) and BP2 (in green), respectively, with loop regions, as well as N- and C-termini also annotated for reference. The two protomers of hNGF are coloured in light and dark grey, respectively. DMSO molecules representative of the two clusters are shown as spheres and colour-coded by element. Figure generated using PyMOL 2.1.0 [30].

Artificial intelligence/machine learning algorithms are extensively employed to enhance the accuracy of protein–ligand interaction prediction [26]. AlphaFold3 [27,28] enhances our ability to model not only single protein structures but also complex biomolecular interactions, including protein–protein interactions, protein–ligand docking, and protein–nucleic acid complexes. Aiming to predict potential DMSO binding site pockets on hNGF, we challenged the advanced AI-based web tool Protenix, an open-source reproduction of AlphaFold3 (https://protenix-server.com/ accessed on 20 May 2025) [29]. Using this tool, we obtained bona fide ab initio models of the hNGF homodimer in complex with DMSO. Supplementary Table S1 reports the ranking scores (including pLDDT, gPDE, pTM, and ipTM) for the best five predicted protein–ligand complexes. Overall, the consistently high values across these metrics pinpoint high-confidence structural predictions. We have thoroughly analyzed the predicted models of the computed hNGF-DMSO complexes. The clustering of the DMSO poses, following the super-imposition of these complexes, highlighted two highly populated ligand-binding pockets (Figure 4).

The binding pockets within 5.0 Å of any atom of the DMSO ligand, labelled as Binding Pocket 1 (BP1) and Binding Pocket 2 (BP2), encompass residues F49, K88, W99, R100, F101, and residues S17, V18, S19, F54, and T56, respectively (Table 1).

A protein-based solution NMR study was carried out to independently identify the structural determinants of the DMSO/hNGF interaction. A titration of hNGF with increasing concentrations of DMSO-*d*_6_ was monitored through 2D ^1^H-^15^N TROSY (Supplementary Figure S3). This experiment allowed the identification of hNGF residues involved in interactions with DMSO, either through direct contact or as a result of structural rearrangements upon DMSO binding.

**Figure 5:**
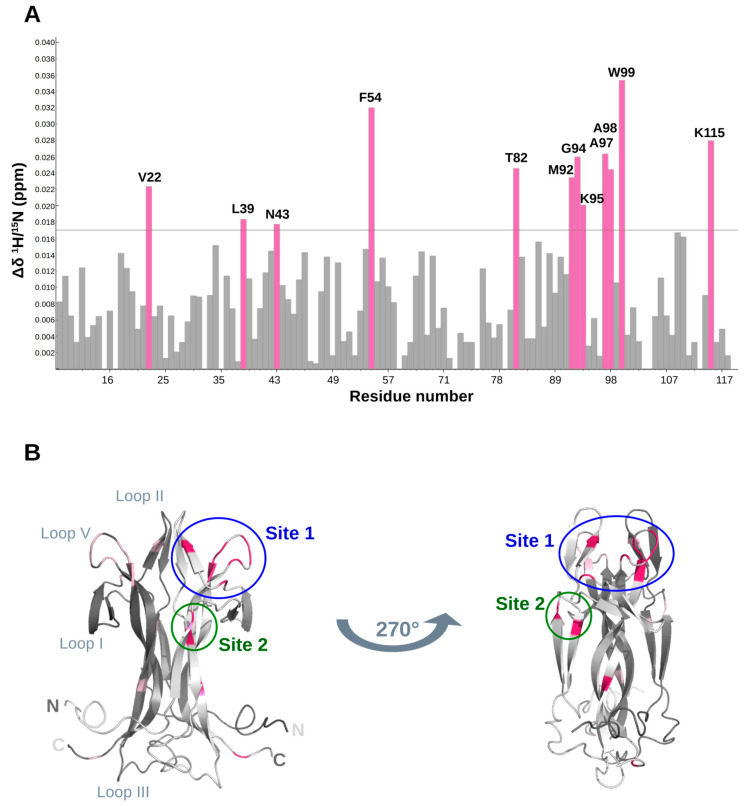
Structural determinants of the hNGF-DMSO interaction. (**A**) DMSO-hNGF interactions by NMR titration. Map of combined ^1^H/^15^N Chemical Shift Perturbations (CSPs, Δδ) of the H_N_ groups of hNGF upon DMSO binding. Residues exhibiting the largest CSP (Δδ > 0.017 ppm: threshold grey line shown in the Figure) are labelled in pink. (**B**) Mapping of CSP data at the endpoint of the DMSO/hNGF titration onto the 3D NMR solution structure of hNGF (PDB ID: 6YW8). Residues with significant CSP (Δδ > 0.017 ppm) are highlighted in pink. The two protomers of hNGF are coloured in light and dark grey, respectively. Two representations of hNGF are shown, with a 270° rotation along the *z*-axis, one with respect to the other. Proposed binding sites are indicated as Site 1 (in blue) and Site 2 (in green). Loop regions, as well as N- and C-termini also annotated for reference. Figure generated using PyMOL 2.1.0 [30].

An analysis of the combined ^1^H/^15^N Chemical Shift Perturbation (CSP, Δδ) showed specific residues likely involved in DMSO binding, showing a combined ^1^H/^15^N CSP (Δδ) greater than 0.017 ppm (Figure 5A), namely, V22, L39, N43, F54, T82, M92, G94, K95, A97, A98, W99, and K115 (Table 1). Overall, there is a large predominance of hydrophobic residues, consistent with the interaction properties of DMSO. Mapping these residues onto the three-dimensional structure of hNGF revealed two main regions of interaction (Figure 5B): the first one (named as Site 1) involves residues from Loop II and V and the second (named as Site 2) is located along the central β-strand of the protein’s main stem. This observation suggests a potential binding of up to four DMSO molecules per hNGF dimer at saturation.

**Figure 6:**
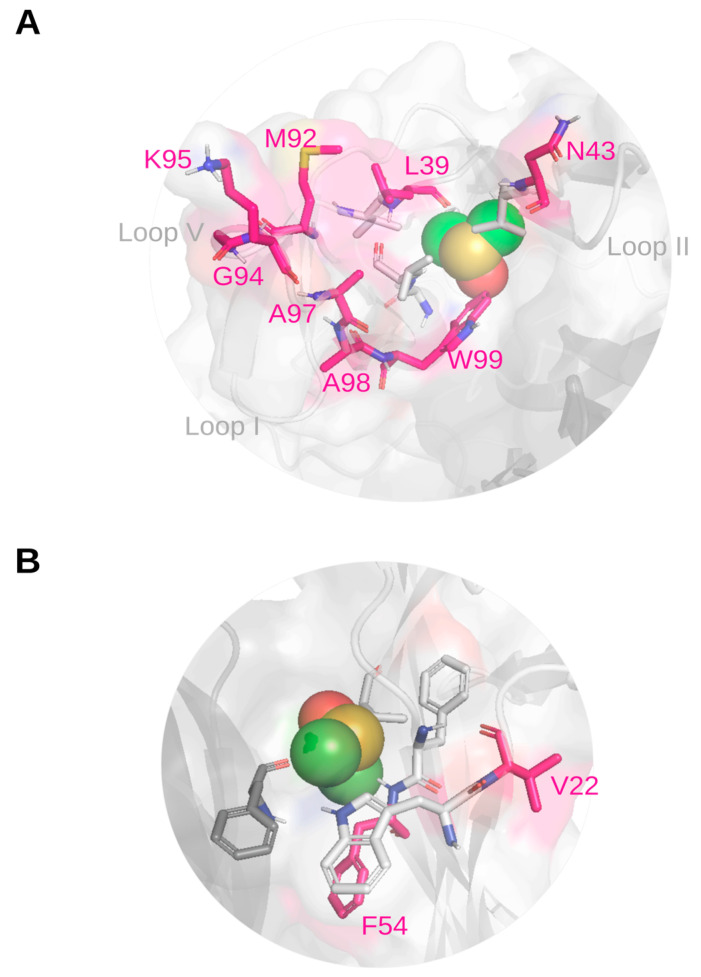
Close-up views of the most significant representative HADDOCK docking poses of DMSO bound to hNGF. (**A**) Top view of the proposed Site 1 identified by CSP analysis, encompassing Loops II and V. (**B**) Side view of the proposed Site 2 identified by CSP analysis, involving the central stem region. The two protomers of hNGF are coloured in light and dark grey, respectively. The protein is displayed in cartoon representation, with an overlaid surface. Residues exhibiting the largest CSPs are shown as pink sticks and colour-coded by element. Hydrophobic residues of the binding site are depicted in dark grey and colour-coded by element. DMSO molecules are represented as spheres and colour-coded by element. Figure generated using PyMOL 2.1.0 [30].

A refined model of the possible orientations of DMSO molecules on the protein was obtained through HADDOCK docking [31,32] by using the residues with the largest combined ^1^H/^15^N CSP (Δδ > 0.017) as active restraints. The resulting DMSO binding poses nicely fit with the experimental NMR CSP data, supporting the proposed interaction sites and binding conformations. HADDOCK grouped 184 structures into seven clusters, representing 92% of the water-refined models. Two representative poses of the top two clusters are reported in Figure 6. The docking results (Table 1) further confirm that DMSO mainly interacts with hNGF through hydrophobic interactions (Figure 6). Specifically, the one DMSO molecule engages V38, L39, V42, N43, A89, L90, M92, A97, A98, and W99 (Site 1), while the other sits in a region involving residues W21, V22, F53, F54, F86, V87 (Site 2).

The HADDOCK protein–ligand complexes guided by the experimental NMR-CSP restraints are notably in close agreement with the biomolecular landscape as highlighted by the bona fide ab initio models of the hNGF-DMSO complex predicted by Protinex (Figure 4). Interestingly, the Protenix (Figure 4) and HADDOCK (Figure 6) models both share residues W99 (BP1 and Site 1) and F54 (BP2 and Site 2), respectively, to be involved in DMSO binding (see Table 1). Remarkably, these two residues are also those that exhibit the largest experimental combined ^1^H/^15^N CSP (Δδ).

**Table 1:** Residues involved in the hNGF-DMSO interaction identified by ab initio Protenix models, NMR CSP, and HADDOCK, guided by the experimental NMR-CSP restraints (left side). Residues involved in the hNGF–small-molecule antagonist interaction identified by computational docking (right side). The residues highlighted in bold are involved in NGF-DMSO shared binding sites according to the experimental and computational approaches.

DMSO					NGF Antagonists			
Protenix BP1 (5 Å)	Protenix BP2 (5 Å)	NMR CSP	HADDOCK Site 1	HADDOCK Site 2	PD 90780/ALE 0540/Ro 08-2750 [33,34]	Y1036/Y1370 [35,36]	PQC 078/PQC 083 [37]	BVNP-0197 [38]
	S17							
	V18							
	S19							
				**W21**		**W21**		
		V22		V22				
						G23		
							D30	
					I31			
					K32		K32	
					K34			
			V38					
		L39	L39					
								E41
			**V42**					**V42**
		**N43**	**N43**					**N43**
								I44
								N45
								N46
								S47
								V48
**F49**								**F49**
				F53				
	F54	F54		F54				
						E55		
	T56							
						K57		
		T82						
				F86				
				V87				
K88								
			A89					
			L90					
		M92	M92					
		G94						
		**K95**			**K95**			
					Q96			Q96
		**A97**	**A97**					**A97**
		**A98**	**A98**					**A98**
**W99**		**W99**	**W99**					**W99**
**R100**					**R100**		**R100**	
**F101**					**F101**		**F101**	
		K115						

## 3. Discussion

Our study presents compelling evidence that DMSO, commonly used as a solvent to solubilize small molecules during early-stage drug discovery, can significantly affect the interpretation of NGF-targeting ligand experiments.

These results emphasize that DMSO’s impact on hNGF’s overall 3D structure is minimal, limited to weak and non-disruptive interactions. Detailed biophysical characterization and structural analyses confirm that the presence of DMSO does not compromise hNGF’s structural integrity, preserving its native conformation and functional properties. Our findings reveal that DMSO interacts with hNGF in an unambiguous manner, characterized by low-affinity binding. Additionally, we demonstrate that the presence of DMSO does not cause any significant changes in hNGF’s interaction with its receptors, either in terms of binding affinity or the kinetics of association and dissociation, indicating that DMSO does not interfere with hNGF’s ability to recognize and bind its physiological partners. These results suggest that hNGF maintains its native binding properties despite DMSO exposure, supporting the notion that the interaction between hNGF and DMSO does not induce conformational alterations that could impact receptor engagement.

While the interaction is weak, it has the potential to modulate hNGF’s ligand-binding capabilities under certain conditions. Detailed analyses shed light on the nature of these DMSO-hNGF interactions, highlighting specific binding sites and the structural dynamics involved. Remarkably, a certain degree of overlap exists between the residues involved in the hNGF-DMSO interaction sites according to our study, and those engaged in the hNGF–small-molecule antagonist interaction identified by computational docking [33,34,35,36,37,38] (Table 1).

Given that DMSO interacts with hNGF at concentrations as low as 0.05% (i.e., 7 mM), which is above the K_D_ of typical low-affinity binders, its presence can influence the binding kinetics and thermodynamics fingerprint by masking the true binding affinity, thus causing difficulties in the determination of the genuine efficacy of small-molecule antagonists targeting hNGF. This interaction with hNGF can lead to misinterpretations, particularly when analyzing low-affinity compounds or antagonists, resulting in overestimated affinities or the erroneous detection of binding site interactions. This underscores the need to carefully control DMSO concentrations in screening assays to avoid confounding results, especially in the early stages of drug discovery when solubility optimization is a critical step.

The importance of these findings cannot be overstated, as they provide critical insights into how solvent–protein interactions, such as those between DMSO and hNGF, can lead to false positives or misestimated affinities in assays designed to identify effective ligands. This knowledge is essential for optimizing the experimental design in hNGF-targeting drug discovery, particularly in the context of pain and inflammation-related conditions. Additionally, by highlighting the potential for solvent interference, this study emphasizes the need for greater caution and methodological rigour in the solubility screening phase, ensuring that DMSO and other solvents are considered integral components of the investigated system when evaluating binding data.

The implications of this research extend beyond hNGF antagonists to broader drug discovery efforts where DMSO is used to dissolve compounds in vitro. Our findings provide a clearer understanding of the biochemical and biophysical effects of solvents on target proteins, which will help to refine screening protocols and improve the reliability of early-stage drug testing. By accounting for these factors, researchers can better identify and validate truly effective hNGF antagonists, facilitating the development of more precise and reliable therapeutics for pain management, inflammation, and neurodegenerative diseases. Thus, this study contributes to the growing body of knowledge on solvent–protein interactions, offering essential guidance for the design of future experiments in drug discovery programs.

## 4. Materials and Methods

### 4.1. Materials

hNGF was recombinantly expressed according to well-established protocols, which exploit in vitro refolding and subsequent proteolytic cleavage of its precursor h-proNGF [39,40]. Expression of h-proNGF VSAR in minimal medium with ^15^N-labelling was carried out as previously optimized [41]. Protein fold was characterized by biophysical techniques (DSF, FT-IR, NMR). ^15^N-ammonium chloride for labelled protein expression was purchased from Eurisotop (Saint-Aubin, France, NLM-467-10).

All chemicals were purchased from MERCK (Darmstadt, Germany, sodium phosphate monobasic dehydrated 71505; sodium phosphate dibasic dehydrated 71643; sodium chloride S9888; Dimethyl sulfoxide D8418; Guanidine hydrochloride G3272; Hepes 54457; Sypro Orange S5692; magnesium chloride M8266; glycine 50046).

### 4.2. Intrinsic Fluorescence

The hNGF solution (15 μg/mL—0.58 µM, with respect to the dimer) was prepared in 50 mM sodium phosphate, a pH of 6.6, 50 mM NaCl, and incubated with increasing DMSO concentrations and with 6 M Guanidine hydrochloride as a control for the denatured protein.

Fluorescence measurements were performed with the Plate Reader Spectrometer Spark (Tecan, Männedorf, Switzerland). The measurements were performed in triplicate using a 324-well plate (Corning, New York, NY, USA), containing 30 μL of protein solution per well. The fluorescence emission spectra were recorded in the wavelength range between 300 and 500 nm, using an excitation wavelength of 280 nm. All spectra were corrected against the corresponding buffer.

### 4.3. Fourier Transform ATR Infrared Spectroscopy (FT-IR)

The FT-IR spectra were acquired using a Vertex 80 spectrometer equipped with a Golden Gate ATR (Bruker, Ettlingen, Germany). Each measurement consisted of 64 scans recorded at 25 °C with a resolution of 4 cm^−1^. hNGF (20 µM, with respect to the dimer) was prepared in 50 mM HEPES, a pH of 7.2, and incubated with an increasing DMSO concentration (from 0.02% to 8%). The subtraction of the scaled blank spectra from the relative sample spectra isolated the amide I and amide II bands, characteristic of the protein.

### 4.4. Differential Scanning Fluorimetry (DSF)

DSF experiments were conducted using a CFX96 Touch Real-Time PCR system (Bio-Rad, Hercules, CA, USA), with excitation and emission wavelengths set at 470–505 nm and 540–700 nm, respectively. hNGF was prepared in 50 mM sodium phosphate buffer with different combinations of pH and NaCl concentrations (pH 6.6 with 50 mM NaCl; pH 6.6 with 150 mM NaCl; pH 7.2 with 50 mM NaCl; pH 7.2 with 150 mM NaCl). For comparison, the same pH and NaCl combinations were also tested using 50 mM HEPES. Samples were mixed to a final hNGF concentration of 8 µM with 20× SYPRO Orange dye, in the presence or absence of increasing DMSO concentrations (0.05%, 0.5%, and 5%, respectively). Fluorescence was monitored as the temperature increased at a rate of 0.2 °C per minute over a range of 20–90 °C. All experiments were performed in duplicate. The melting temperatures (Tm) were determined as the inflection points of the thermal transition curves, calculated using the Boltzmann Sigmoid fit model.

### 4.5. Grating Coupled Interferometry (GCI)

The experiments were conducted using the Creoptix WAVE-delta system (Malvern Pananalytical, Malvern, UK). Materials for the conditioning and amine coupling were purchased from Xantec (Düsseldorf, Germany, amine coupling kit, code K AN-50I). A borate buffer (100 mM sodium borate, pH 9.0, 1 M NaCl) was used for conditioning 4PCH WAVE sensor chips, which feature a quasiplanar polycarboxylate surface (Malvern).

To quantitatively assess hNGF interaction with DMSO, hNGF (30 μg/mL in sodium acetate, pH 5) was immobilized via amine coupling according to the manufacturer’s protocol, achieving a final surface density of 5600 pg/mm^2^. WaveRAPID experiments [42] were conducted at 25 °C using the “weak binder” configuration. In this setup, 10 mM DMSO (corresponding to 0.071% concentration) was injected at a constant flow rate of 400 μL/min in running buffer (50 mM sodium phosphate, pH 6.6, 50 mM NaCl). The association and dissociation phases were set to 5 and 20 s, respectively.

To evaluate the impact of DMSO on NGF’s interaction with its receptors, human TrkA-Fc (R&D, TrkA Fc Chimera, 175-TK-050) (20 μg/mL in sodium acetate, pH 5) and human p75^NTR^-Fc (R&D, R/TNFRSF16 Fc Chimera, 367-NR-050) (50 μg/mL in sodium acetate, pH 5) were immobilized via amine coupling following the manufacturer’s protocol, reaching final surface densities of 3500 pg/mm^2^ and 3000 pg/mm^2^, respectively. WaveRAPID experiments were performed at 25 °C using the “intermediate binder” configuration. hNGF (0.2 μM for TrkA and 1 μM for p75^NTR^) was injected at a constant flow rate of 100 μL/min in running buffer (PBS supplemented with 0.05% P20), both in the absence and presence of 7 mM (0.05%) DMSO. The association and dissociation phases were set to 25 and 300 s, respectively. Regeneration was carried out using two sequential pulses of 10 mM glycine, with a pH of 1.5.

Data analysis and visualization were performed using the WAVEcontrol software 4.0. Corrections, including X and Y offsets, DMSO calibration, and double referencing, were applied during analysis. Kinetic parameters were calculated using the Direct Kinetics engine and a one-to-one binding model. The experiment was conducted at least in duplicate to ensure reproducibility and reliability.

### 4.6. Nuclear Magnetic Resonance (NMR)

Two-dimensional ^1^H-^15^N TROSY spectra were recorded at 30 °C at a Bruker Avance Neo 800 MHz spectrometer (Bruker, Ettlingen, Germany) equipped with a ^1^H/^13^C/^15^N cryoprobe, with 1024 data points in t2, 48 scans, 92 complex points in t1, and a relaxation delay of 1.5 s. The ^1^H and ^15^N sweep widths were 11,161 and 2269 Hz, respectively. Samples contained ^15^N-labelled hNGF (0.1 mM) in 50 mM sodium phosphate at pH 6.6, 50 mM NaCl, and 10% D_2_O. DMSO-*d*_6_ stock solution was prepared in the same hNGF buffer.

For the titration experiment, subsequent DMSO-*d*_6_ additions to the hNGF were performed, from 0.2% to 4%. All spectra were processed using TopSpin 3.6.2 and analyzed using CCPNmr v3.1.1 [43,44].

### 4.7. Computational Methods

Predictions by Protenix [29] were performed without considering any homologous experimental template. The FASTA sequence of hNGF as extracted by the PDB ID 6YW8 NMR solution structure [41] was provided as input, with ten as the number of recycles. Protenix was instructed to generate eight binding poses for the DMSO ligand within each of the five predicted protein models. The DMSO ligand was defined by using a SMILES string [45]. Quality scores were evaluated as reported [46,47].

The docking of DMSO on hNGF was performed using the HADDOCK 2.4 web tool (high ambiguity driven protein–protein docking) (https://rascar.science.uu.nl/haddock2.4/ accessed on 9 May 2025) [31,32]. The docking utilized the solution NMR hNGF 3D structure (PDB ID: 6YW8) and DMSO as ligand. For the docking, the residues with the largest combined ^1^H/^15^N CSP (Δδ > 0.017 ppm) from the NMR study were used as restraints.

## 5. Conclusions

The study of hNGF and its antagonists is pivotal in addressing a wide spectrum of small-molecule ligands targeting pathological conditions, from chronic pain to neurodegenerative diseases.

By exploring the biophysical and structural effects of solvents like DMSO on hNGF interactions, our study provides critical insights into the challenges and opportunities in the early-stage development of small-molecule ligands. Our findings contribute to a better understanding of DMSO’s solubilizing properties by highlighting that it can directly interact with hNGF. However, this interaction does not alter hNGF’s receptor-binding dynamics, suggesting that DMSO does not interfere with hNGF’s functional engagement with its physiological partners. This underscores the importance of understanding solvent–protein interactions, as they can significantly impact the accuracy of experimental outcomes and the development/screening of therapeutic agents.

The broader implications of this work are substantial. hNGF-targeting small molecules represent a promising alternative to monoclonal antibodies, offering advantages in terms of oral bioavailability, cost-effectiveness, and reduced immunogenicity. The elucidation of hNGF’s behaviour in the presence of DMSO provides a foundational framework for optimizing drug design and screening methodologies, ensuring the development of potent and selective antagonists. This is particularly crucial in light of the therapeutic potential of the inhibition of the hNGF function, not only for pain management but also for conditions characterized by inflammation and neurodegeneration.

Moreover, this study highlights the necessity of integrative advanced computational and biophysical tools to refine drug discovery processes. By leveraging experimental tools such as FT-IR, DSF, GCI, and NMR, this research demonstrates the ability to uncover nuanced interactions that might otherwise be overlooked. This comprehensive approach not only boosts our understanding of hNGF’s pharmacology but also sets a precedent for future investigations into neurotrophin-targeting therapies.

In conclusion, this work bridges a critical gap in the field of hNGF research, emphasizing the dual importance of addressing technical challenges in drug development and advancing therapeutic strategies for NGF-related disorders. Although challenges remain, such as achieving specificity and minimizing adverse effects, continued research and innovation in this area are likely to yield cost-effective, patient-friendly alternatives to monoclonal antibody therapies. The careful consideration of solvent effects, such as those caused by DMSO, is critical to advancing this field. Therefore, this study serves as a crucial step toward realizing the full potential of hNGF antagonists in clinical applications.

## Data Availability

The original contributions presented in this study are included in the article/Supplementary Materials. Further inquiries can be directed to the corresponding author.

## Supplementary Materials

The following supporting information can be downloaded at: https://www.mdpi.com/article/10.3390/molecules30143030/s1, Figure S1: FT-IR spectrum of DMSO; Figure S2: Impact of DMSO concentration on hNGF melting curves obtained by DSF; Figure S3: NMR titration of DMSO to hNGF. Table S1: Protenix confidence scores for assessing the reliability of the predictions of the Protenix ab initio hNGF-DMSO complexes.
