# Supplementary material for: Unveiling the Solvent Effect: DMSO Interaction with Human Nerve Growth Factor and Its Implications for Drug Discovery
Source: Molecules. 2025 Jul 19;30(14):3030. doi: 10.3390/molecules30143030 (PMC12300743; doi:10.3390/molecules30143030)
Supplement: Supplementary file 1 [file molecules-30-03030-s001.zip › molecules-3719945-supplementary.pdf]

Supporting Material

Unveiling the Solvent Effect: DMSO Interaction with Human Nerve Growth Factor and Its Implications for Drug Discovery

Francesca Paoletti [1], Tjaša Goričan [2], Alberto Cassetta [1], Jože Grdadolnik [2], Mykola Toporash [1], Doriano Lamba [1,3], Simona Golič Grdadolnik [2] and Sonia Covaceuszach [1,*]

1 Institute of Crystallography-C.N.R.-Trieste Outstation, 34149 Trieste, Italy; francesca.paoletti@cnr.it (F.P.); alberto.cassetta@cnr.it (A.C.); mykolatoporash@cnr.it (M.T.); doriano.lamba@cnr.it (D.L.)
2 Laboratory for Molecular Structural Dynamics, Theory Department, National Institute of Chemistry,
1001 Ljubljana, Slovenia; tjasa.gorican@ki.si (T.G.); joze.grdadolnik@ki.si (J.G.); simona.grdadolnik@ki.si (S.G.G.)
3 Interuniversity Consortium "Biostructures and Biosystems National Institute", 00136 Rome, Italy
* Correspondence: sonia.covaceuszach@cnr.it

Abstract

Background: Nerve Growth Factor (NGF) is essential for neuronal survival and function and represents a key therapeutic target for pain and inflammation-related disorders, as well as for neurodegenerative diseases. Small-molecule antagonists of human NGF (hNGF) offer advantages over monoclonal antibodies, including oral availability and reduced immunogenicity. However, their development is often hindered by solubility challenges, necessitating the use of solvents like dimethyl sulfoxide (DMSO). This study investigates whether DMSO directly interacts with hNGF and affects its receptor-binding properties. Methods: An integrative/hybrid computational and experimental biophysical approaches were used to assess DMSO-NGF interaction, by combining machine-learning tools and Nuclear Magnetic Resonance (NMR), Fourier Transform Infrared (FT-IR) spectroscopy, Differential Scanning Fluorimetry (DSF) and Grating-Coupled Interferometry (GCI). These techniques evaluated binding affinity, conformational stability, and receptor-binding dynamics. Results: Our findings demonstrate that DMSO binds hNGF with low affinity in a specific yet non-disruptive manner. Importantly, DMSO does not induce significant conformational changes in hNGF nor affect its interactions with its receptors. Conclusions: These results highlight the importance of considering solvent-protein interactions in drug discovery, as these low affinity, yet specific, interactions can affect experimental outcomes and potentially alter the small molecules binding to the target proteins. By characterizing DMSO-NGF interactions, this study provides valuable insights for the development of NGF-targeting small molecules, supporting their potential as effective alternatives to monoclonal antibodies for treating pain, inflammation, and neurodegenerative diseases.

Table of Contents:

Figure S1. FT-IR spectrum of DMSO in HEPES buffer after buffer subtraction.

Figure S2. Impact of DMSO concentration on hNGF melting curves obtained by DSF.

Figure S3. NMR titration of DMSO to hNGF.

Table S1. **Protenix confidence scores for assessing the reliability of the predictions of the hNGF-DMSO complexes**.

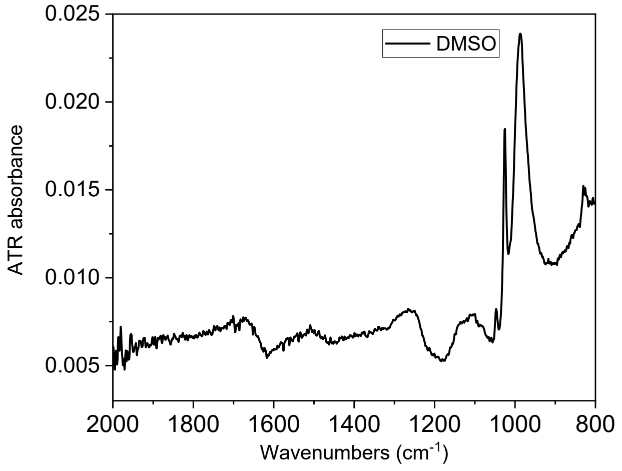

Figure S1. – FT-IR spectrum of DMSO in HEPES buffer after buffer subtraction.

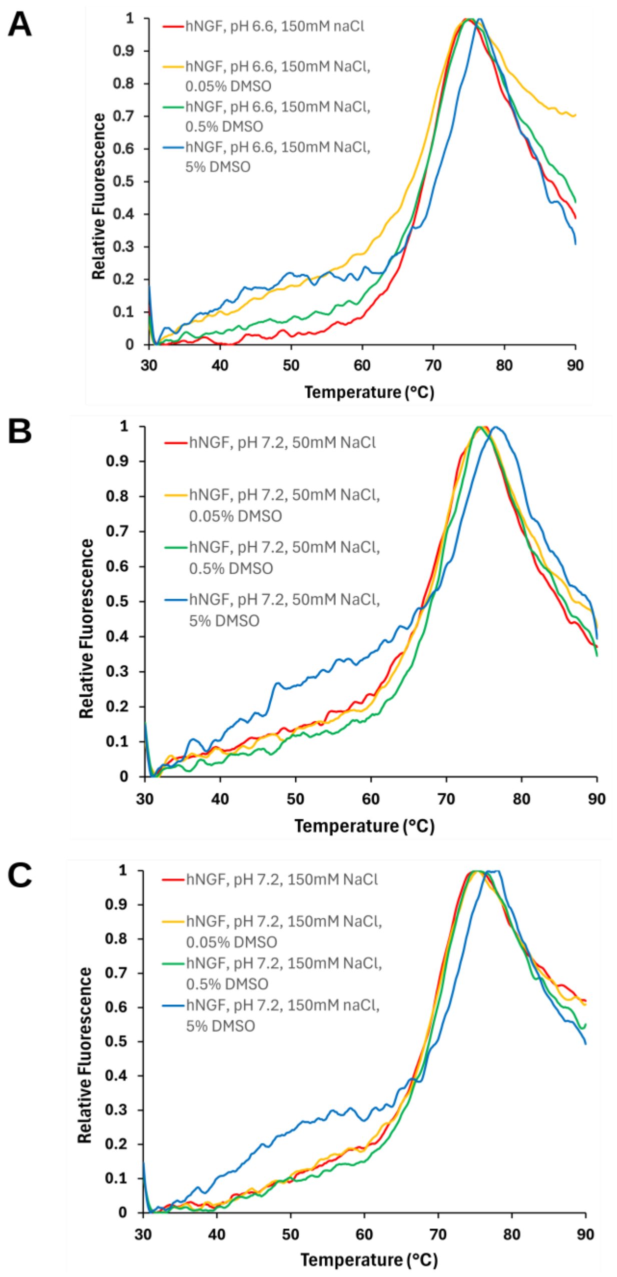

Figure S2 - Impact of DMSO concentration on hNGF melting curves obtained by DSF. Normalized curves are shown. Different combination of pH and salt are shown: **A** - sodium phosphate 50 mM pH 6.6 and 150 mM NaCl. Tm shifts: 69.0±0.5 °C at 0% DMSO; 68.0±1.1 °C at 0.05% DMSO; 69.0±0.7 °C at 0.5% DMSO; 70.2±0.1 °C at 5% DMSO. **B** - sodium phosphate 50 mM pH 7.2 and 50 mM NaCl. Tm shifts from 66.7±0.2 °C at 0% DMSO; 67.0±0.3 °C at 0.05% DMSO; 67.9±0.1 °C at 0.5% DMSO; 68.9±1.1 °C at 5% DMSO. **C** - sodium phosphate 50 mM pH 7.2 and 150 mM NaCl. Tm shifts from 68.1±0.2 °C at 0% DMSO; 68.4±0.1 °C at 0.05% DMSO; 69.4±0.9 °C at 0.5% DMSO; 69.5±0.5 °C at 5% DMSO. In each experiment, the effect of three different DMSO concentration were tested (0.05 % - yellow line, 0.5 % - green line, 5% - blue line).

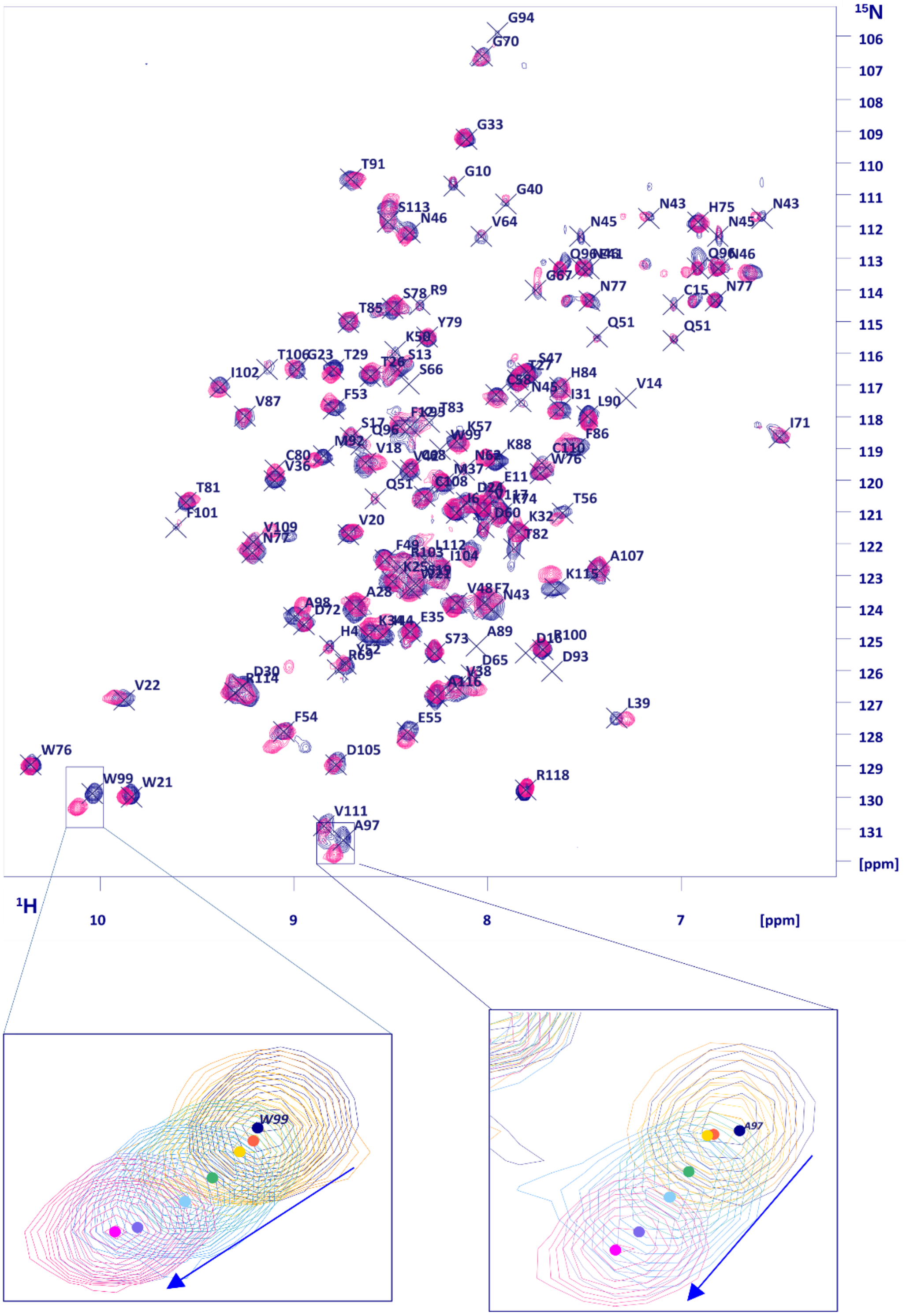

Figure S3. NMR titration of DMSO to hNGF. Top: overlay of 2D ^{1}H-^{15}N HSQC spectra of 0.1 mM hNGF at 0% DMSO (dark blue); 0.2% DMSO (red); 0.4% DMSO (yellow); 0.6% DMSO (green); 1% DMSO (cyan); 2% DMSO (violet) and at 4% DMSO (magenta). Assigned backbone amide resonances are shown. Spectra were collected at 30°C and 800 MHz. Bottom: expanded regions of 2D ^{1}H-^{15}N HSQC spectra showing an example of larger shift of the residues A97 and W99 during titration. The center of each peak is marked with a dot of the same color as the corresponding peak levels.

Model	pLDDT[a]	gPDE[b]	pTM[c]	ipTM[d]	Rankig Score[e]
**1**	92.5	0.581	0.949	0.936	0.938
**2**	92.5	0.574	0.950	0.935	0.938
**3**	92.5	0.594	0.948	0.932	0.935
**4**	92.5	0.606	0.946	0.929	0.933
**5**	93.0	0.607	0.945	0.927	0.930

Table S1. **Protenix confidence scores for assessing the reliability of the predictions of the hNGF-DMSO complexes**.

[a]The predicted Local Distance Difference Test (pLDDT) measures the confidence in the local structure, estimating how well the prediction would agree with an experimental structure. It is based on the local distance difference test Cα (lDDT-Cα), which is a score that does not rely on superposition but assesses the correctness of the local distances (**S1**).

[b]Global Predicted Distance Error (PDE) score measures the expected error in inter-residue distances. It measures the difference between the predicted and actual distances between residues in a protein. It assesses how accurately a model predicts the 3D structure of a protein. Lower values indicate greater confidence.

[c]pTM is an integrated measure of how well Protinex has predicted the overall structure of the complex. It is the predicted Template Modelling (TM) score for a superposition between the predicted structure and the hypothetical true structure. A TM score above 0.5 means the overall predicted fold for the complex might be similar to the true structure (**S2**).

[d]ipTM measures the accuracy of the predicted relative positions of the subunits forming the protein-protein complex, namely the hNGF homo-dimer. Values higher than 0.8 represent confident high-quality predictions.

[e]Predicted confidence score for ranking complexes. Higher values indicate greater confidence.
